# The level of leisure time physical activity is associated with work ability-a cross sectional and prospective study of health care workers

**DOI:** 10.1186/1471-2458-13-855

**Published:** 2013-09-17

**Authors:** Elin Arvidson, Mats Börjesson, Gunnar Ahlborg, Agneta Lindegård, Ingibjörg H Jonsdottir

**Affiliations:** 1 The Institute of Stress Medicine, Carl Skottbergs gata 22 B, 413 19 Gothenburg, Sweden; 2 Swedish School of Sports & Health Science and the Department of Cardiology, Karolinska University Hospital, Stockholm, Sweden; 3 Centre for person-centred care (GPCC), Gothenburg University, Gothenburg, Sweden; 4 The Department of Public Health Community Medicine, Sahlgrenska Academy, University of Gothenburg, Gothenburg, Sweden; 5 The Department of Neuroscience and Physiology, Sahlgrenska Academy, University of Gothenburg, Gothenburg, Sweden

**Keywords:** Longitudinal study, Predictive instrument, Work ability index

## Abstract

**Background:**

With increasing age, physical capacity decreases, while the need and time for recovery increases. At the same time, the demands of work usually do not change with age. In the near future, an aging and physically changing workforce risks reduced work ability. Therefore, the impact of different factors, such as physical activity, on work ability is of interest. Thus, the aim of this study was to evaluate the association between physical activity and work ability using both cross sectional and prospective analyses.

**Methods:**

This study was based on an extensive questionnaire survey. The number of participants included in the analysis at baseline in 2004 was 2.783, of whom 2.597 were also included in the follow-up in 2006. The primary outcome measure was the Work Ability Index (WAI), and the level of physical activity was measured using a single-item question. In the cross-sectional analysis we calculated the level of physical activity and the prevalence of poor or moderate work ability as reported by the participants. In the prospective analysis we calculated different levels of physical activity and the prevalence of positive changes in WAI-category from baseline to follow-up. In both the cross sectional and the prospective analyses the prevalence ratio was calculated using Generalized Linear Models.

**Results:**

The cross-sectional analysis showed that with an increased level of physical activity, the reporting of poor or moderate work ability decreased. In the prospective analysis, participants reporting a higher level of physical activity were more likely to have made an improvement in WAI from 2004 to 2006.

**Conclusions:**

The level of physical activity seems to be related to work ability. Assessment of physical activity may also be useful as a predictive tool, potentially making it possible to prevent poor work ability and improve future work ability. For employers, the main implications of this study are the importance of promoting and facilitating the employees’ engagement in physical activity, and the importance of the employees’ maintaining a physically active lifestyle.

## Background

The cause of many diseases today is an unhealthy lifestyle, of which physical inactivity is one aspect [1]. The positive effects of physical activity (PA) are well known, and PA plays an important role in primary and secondary prevention of disorders such as obesity, type 2 diabetes, and cardiovascular diseases [2-5]. An increasing number of studies have reported that mental health complaints can also be affected by PA [6,7].

It has been shown that with increasing age, physical capacity decreases, and the need and time for recovery increases [1,8-10]. At the same time, the demands of work usually do not change with age. Thus, an aging and physically changing workforce which risks reduced work ability but faces the same occupational demands may be adversely affected by these processes in future working life. This effect may be aggravated by increasing levels of psychosocial stress and insufficient recovery [10]. A recent study by Von Bonsdorff et al. found that frequent emotional exhaustion, low job control and poor work ability increased the prevalence of thoughts of early retirement among aging workers [11].

Work ability may be seen as a balance between the demands of work and the individual’s resources. The comprehension of the term work ability varies somewhat depending on the perspective in which the term is used. In one context, it may be a question of productivity, and in another, it may be a matter of the physical capacity to perform the tasks given [12]. In previous research, a number of different methods have been used to asses work ability. One of them is the well-established self-reporting questionnaire the Work Ability Index (WAI): a valid and reliable instrument for assessing work ability [13-18].

A number of prospective studies have evaluated possible predictors of work ability [19-21], but the role of PA in this regard is still not fully known. In view of the intentions of these studies, there is a clear need for a simple tool to predict an individual’s work ability. Such a tool might increase the likelihood of finding individuals at risk of decreased work ability, and thereby enable early preventive intervention.

Thus, the aim of this study was to investigate whether PA could help to predict future work ability by studying the relationship between self-reported level of leisure time PA and self-reported work ability at baseline, and at a two year follow-up. We aimed to explore 1) if there was a cross-sectional relationship between PA and work ability, and 2) if the level of PA at baseline could predict work ability over time, in a large cohort of health care personnel in Western Sweden.

## Methods

### Study population

This study is part of a larger survey conducted in 2004 with a follow-up in 2006. The general aim of the original study was to investigate different aspects of stress and stress-related health in the public service sector. A randomly selected sample of 6.000 persons from the employee registers at Region Västra Götaland (n = 5.300 out of 48.600 employees), mainly healthcare workers (82%), and workers at the Social Insurance Offices (n = 700 out of 2.200 employees) in the same geographical region of Western Sweden, were asked to participate. Only employees with duration of employment of at least one year and at least 50% of full-time employment were included. The participants received a questionnaire in the post, and the response rate after two reminders was 61%; thus a total of 3.717 subjects responded at baseline. The respondents were then asked to participate in a follow-up two years later, and this time the response rate was 85%. In total, 2.783 individuals (2.398 women and 385 men) responded both at baseline and at follow-up and provided complete data for all the variables used in the analyses at baseline. Of these, 2.597 individuals were also included in the prospective analysis (drop out n = 186 due to missing data). There were no significant differences between individuals with missing data at follow-up in terms of age, sex, BMI, WAI and level of PA compared with participants with complete data. The study was approved by the Regional Ethical Review Board, Gothenburg, Sweden and conducted in accordance with the 1964 Declaration of Helsinki. By completing and sending in the questionnaire, the participants gave their consent to participation.

### Assessment of physical activity

The subjects rated their PA according to a single-item question developed by Saltin and Grimby [22]. It evaluates the level of PA during leisure time, and has been widely used in epidemiological studies [23-27]. This tool has been shown to discriminate between sedentary and active counterparts regarding maximal oxygen uptake [28] and has been validated against biological measures [29]. It has also been found to identify individuals with increased cardiovascular risk factors [30]. The participants reported one level (1–4) that best corresponded to their PA during the preceding three months; 1) mostly sedentary (S), 2) light PA (such as walking, gardening or bicycling to work) at least two hours a week (LPA), 3) more intensive exercise (such as doing aerobics, dancing, swimming, playing football or heavy gardening) at least two hours a week (MPA), or 4) high intensity exercise several times per week, at least five hours (VPA).

### Assessment of work ability

To assess work ability, the self-report instrument Work Ability Index (WAI) was used [13,14]. It was developed in Finland in the early 1980s [13] with the aim of investigating how long an employee was able to work before retirement and the extent to which the ability to work was influenced by the content and demands of work. WAI has been validated against both clinically assessed factors and cardio-respiratory capacity [31,32]. WAI consists of seven items regarding both physical and psychological aspects of work ability. The score ranges from 7 to 49, and four categories of work ability are defined as follows; the minimum score of 7–27 indicates “poor” work ability, 28–36 “moderate” work ability, 37–43 “good” work ability and 44–49 “excellent” work ability [14]*.*

### Data analysis and statistics

Descriptive statistics are given in terms of counts and percentages for categorical variables, and means and standard deviations (SD) for continuous variables. Pearson’s chi-square test was used to analyse group differences at baseline, and the level of significance was set at p < 0.05. Only participants with complete data on all variables used in the analyses were included.

In the cross-sectional analysis we calculated the level of PA and the prevalence of reporting poor or moderate work ability, with sedentary individuals as the reference group. In the prospective analysis we calculated different levels of PA and the prevalence of positive changes in WAI-category from baseline to follow-up. Participants who changed 1) from “poor” to “moderate”, “good” or “excellent”, 2) from “moderate” to “good” or “excellent” or 3) from “good” to “excellent” and 4) participants who maintained the WAI-category “excellent” were classified as having a positive change.

In both the cross-sectional and the prospective analyses, data was analysed using the log binomial model, which is a generalized linear model with a logarithmic link function and a binomial distribution function, and expressed as prevalence ratios (PR) with 95% confidence intervals (CI). Adjustments were made for age, sex and body mass index (BMI).

## Results

### Baseline characteristics

The mean age at baseline was 47 years (22–70 years), and the mean BMI of the participants was 25 (SD 3.6). More than half of the participants reported LPA, one third reported MPA or VPA and 14% reported being mostly sedentary. One fifth reported poor or moderate work ability while just over 80% of the participants reported good or excellent work ability (Table 1).

**Table 1:** Baseline characteristics of the participants, 2004 (n = 2.783)

	**Baseline**
**Age**; mean (SD)	47 (10)
**BMI**^1^; mean (SD)	24.6 (3.6)
**Sex**; n (%)	
Women	2.398 (86)
Men	385 (14)
**Pysical activity-level**; n (%)	
Sedentary	396 (14)
Light physical activity	1.474 (53)
Moderate physical activity	843 (30)
Vigorous physical activity	70 (3)
**WAI-category**; n (%)	
Poor or moderate	507 (19)
Good or excellent	2.276 (81)

^1^BMI = Body Mass Index.

### Cross-sectional analysis of work ability and physical activity

The number of participants reporting poor or moderate work ability at baseline was 507. The analysis showed that with an increased level of PA, using participants in the sedentary group as a reference, the prevalence of individuals reporting poor or moderate work ability decreased. Adjustments for age, sex and BMI did not change the estimates considerably and the result was still statistically significant (p < .001) (Table 2).

**Table 2:** Cross sectional analysis showing the likelihood of reporting poor or moderate work ability for different levels of physical activity at baseline in 2004, presented as prevalence ratios (PR) and 95% confidence interval (CI)

	**Numbers included in the analysis n (%)**	**Unadjusted**		**Adjusted**^**1**^	
		**PR**	**95% CI**	**PR**	**95% CI**
Sedentary^2^	119 (24)	1		1	
Light physical activity	291 (57)	0.66	0.55-0.79	0.68	0.57-0.81
Moderate physical activity	94 (19)	0.37	0.29-0.47	0.43	0.34-0.55
Vigorous physical activity	3 (1)	0.14	0.05-0.44	0.19	0.06-0.59

^1^Adjusted for age, sex and body max index.

^2^Reference group.

### Physical activity as a predictor of work ability at follow-up

The number of individuals with a positive change in WAI-category between 2004 and 2006 was 1.157. Using participants in the sedentary group as a reference, individuals reporting LPA, MPA or VPA were progressively more likely to have improved their work ability or maintained excellent work ability during the two-years prior to the follow-up. Adjustments for age, sex and BMI did not change the estimates considerably and the result was still statistically significant (p < .001) (Table 3).

**Table 3:** **Prospective analysis showing the likelihood of having a positive change in work ability index**^**1 **^**from 2004 to 2006 for different levels of physical activity reported at baseline in 2004 (n = 2.597), presented as prevalence ratios (PR) and 95% confidence interval (CI)**

	**Numbers included in the analysis n (%)**	**Unadjusted**		**Adjusted**^**2**^	
		**PR**	**95% CI**	**PR**	**95% CI**
Sedentary^3^	120 (10)	1		1	
Light physical activity	568 (49)	1.27	1.08-1.49	1.26	1.07-1.48
Moderate physical activity	431 (37)	1.64	1.40-1.93	1.57	1.33-1.84
Vigorous physical activity	38 (3)	1.73	1.34-2.23	1.59	1.23-2.06

^1^Change in the positive direction between the four categories “poor”, “moderate”, “good” and “excellent”, or remaining in the category “excellent”.

^2^Adjusted for age, sex and BMI.

^3^Reference group.

## Discussion

The main result of this study is that self-reported leisure-time PA was positively related to work ability in both cross-sectional and prospective analyses. In the prospective analysis even LPA seemed to increase the chance of having improved work ability, and higher levels of PA (MPA or VPA) was even more strongly related to a positive change in WAI. The clinical implication of this study is that in addition to the recommended amount of daily PA (30 minutes of moderately intense PA) any activity above this level could further improve work ability.

The results of the present study support the value of a physically active work force. In the near future the proportion of older workers (aged 50 years or above) will increase in comparison to the number of younger workers (aged up to 24 years) in many countries [33]. It must be of interest, not only for the individual employees, but also for employers and society at large, to promote and maintain a good working life and sustained work ability in order to keep a stable workforce in the future. It has previously been found that poor or moderate work ability increases the risk of early retirement [34]. It has also been shown that the better the work ability index is, the later the retirement takes place [35]. In addition to the obvious financial advantages, Seitsamo et al. found that individuals in an older working population (mean age 51) who maintained good work ability also remained active, both physically and with hobbies, and were more satisfied with their lives [36].

A review concerning workplace PA interventions showed positive effects of factors such as PA behaviour, fitness, work attendance and work ability [37]. In a work place intervention study, Pohjonen et al. reported a slower decline in the Work Ability Index score of an intervention group performing PA during the work day twice a week, for a period of nine months, compared to a control group, at a five year follow-up. The decrease in work ability was three times faster in the control group than in the intervention group [19]. In contrast to our study, Pohjonen et al. used a decrease in the WAI-index as the outcome measure. We preferred to use a salutogenic approach, by focusing on positive changes in WAI-category between the baseline and the follow-up, but the results from both studies still point in the same direction. A range of factors that increase the possibility of reaching a sustainable change in PA behaviour are identified. Among them are motivational support and the importance of finding a purposeful activity [38]. For employers it may be cost-effective to facilitate an active lifestyle through workplace interventions. However, initiatives taken by employers to promote a healthy lifestyle among employees should never replace any warranted improvements of the work environment.

In this study we also showed that a single item question for self-reported level of PA was able to serve as a predictive tool for work ability. In van den Berg’s review from 2008, three of the studies using a longitudinal design evaluated different methods of predicting work ability [20,21,39]. One of the studies found that functional, postural and perceived balance tests were able to predict work ability in fire fighters [21]. Another study found that fitness tests could predict work ability in home care workers, and yet another study showed that specific factors of work load and life style could predict individual variations in work ability [20,39]. Self-reported PA has previously been used to predict, for example, body composition in children, body density in women and cardiovascular disease [40-42]. However, to our knowledge, no previous study has studied self-reported PA as a predictor of work ability. Since it is also a relatively simple instrument, it could be found to be useful, particularly in occupational health settings. This study shows that PA is of great importance for the development of work ability over time, and that the level of PA seems to be able to predict future work ability at least two years ahead. The single-item question used in our study makes it possible to find individuals who are potentially in the risk zone of deteriorating work ability. Thus, self-perceived level of PA can be applied both as a preventive and as a health-promoting tool, which may aid the efforts to increase individual performance as well as decreasing work absenteeism.

### Limitations

There are several limitations to this study which should be mentioned. Using work ability as an outcome measure in research studies is relatively new and complex. Depending on the definition, there are different methods to evaluate work ability. In the 2008 interim report of the work-ability investigation of the Swedish government, work ability was divided into three dimensions; a physical, a psychological and a social dimension. The interaction between these factors and the demands of the work determine the work ability [43]. The WAI is a multidimensional scale producing a total score which is not always easily interpreted. We chose this measure since it is frequently used and has been constructed and validated in a Scandinavian context.

In this study, the level of PA is based only on self-reported leisure time PA. In a study concerning the significance of level of PA on work ability, it may seem relevant to also consider the time spent in PA during work. It may be argued that an individual with a high level of activity during the work day has a greater need for recovery during leisure time than an individual with sedentary work. However, the self-assessed leisure time PA measure used in this study has been shown to be associated with the overall fitness level of the individual [28]. In addition, our study population of health care personnel is rather homogenous in terms of PA during work, i.e. a relatively active work situation compared to many other types of jobs. Therefore, the results indicate that even in a population with a comparatively high level of PA during work, the activity during leisure time makes a difference with regard to self-reported work ability.

Another limitation is the generalizability of our data, since mainly health care workers are included in the study. Therefore, we can not conclude whether the same relationship would be found in other working populations.

Maintaining good work ability is an important issue for every employee during his or her entire working life. In this perspective, a follow-up time of two years is short. Since this study was based on an extensive survey with follow-ups even after four and six years as well, there will be an opportunity to elucidate whether the result of this study will remain significant in a longer perspective. However, in this type of research, there is always a potential risk for regression to the mean. We have tried to reduce the effect by the inclusion of individuals maintaining excellent work ability, and thereby diminish the latent risk to some extent. An additional analysis was made also with the negative change in work ability index. The result remained unaffected and we thus chose to focus only on the positive change.

## Conclusion

The level of PA seems to be related to work ability. Assessments of PA may also be useful as a predictive tool, potentially enabling the prevention of poor work ability and to improving future work ability. The main implications of this study for employers are the importance of promoting and facilitating the employees’ engagement in PA and in the importance of the employees maintaining a physically active lifestyle.

## Abbreviations

PA: Physical activity; LPA: Light physical activity; MPA: Moderate physical activity; VPA: Vigorous physical activity.

## Competing interests

The authors declare that they have no competing interests.

## Authors’ contributions

GA, IJ and ALA contributed to the data collection. All authors were involved in formulating the research question and planning the data analysis. EA performed the statistical analysis and wrote the manuscript with the assistance of the other authors, all of whom discussed the results and commented on the manuscript. All authors read and approved the final manuscript.

## Pre-publication history

The pre-publication history for this paper can be accessed here:

http://www.biomedcentral.com/1471-2458/13/855/prepub
